# Why is interoperability between the two fields of chemical crystallography and protein crystallography so difficult?

**DOI:** 10.1107/S2052252519010972

**Published:** 2019-08-13

**Authors:** Alice Brink, John R. Helliwell

**Affiliations:** a Department of Chemistry, University of the Free State, Nelson Mandela Drive, Bloemfontein, Free State 9301, South Africa; b Department of Chemistry, University of Manchester, Brunswick Street, Manchester M13 9PL, UK

**Keywords:** interoperability, chemical crystallography, macromolecular crystallography, Cambridge Structural Database, Protein Data Bank

## Abstract

The interoperability of chemical and biological crystallographic data is a key challenge to research and its application to pharmaceutical design. Research attempting to combine data from the two disciplines, small-molecule or chemical crystallography (CX) and macromolecular crystallography (MX), will face unique challenges including variations in terminology, software development, file format and databases which differ significantly from CX to MX. This perspective overview spans the two disciplines and originated from the investigation of protein binding to model radiopharmaceuticals. The opportunities of interlinked research while utilizing the two databases of the CSD (Cambridge Structural Database) and the PDB (Protein Data Bank) will be highlighted. The advantages of software that can handle multiple file formats and the circuitous route to convert organometallic small-molecule structural data for use in protein refinement software will be discussed. In addition some pointers to avoid being shipwrecked will be shared, such as the care which must be taken when interpreting data precision involving small molecules versus proteins.

## Abstract 2

This is a perspective overview of the opportunities and challenges encountered when working at the interface of chemical (CX) and macromolecular crystallography (MX) as a result of differences in file formats and nomenclature between these sister disciplines. It utilizes the two premier databases in crystallography, the Cambridge Structural Database and Protein Data Bank in the research and development of pharmaceutical and medical imaging agents, the latter being of specific interest to the authors. The interdisciplinary obstacles between CX and MX, where data should be FAIR, *i.e.* findable, accessible and interoperable and reusable, are specified.

## 1. Introduction   

Crystallography examines the arrangement of atoms in crystalline solids, for the purpose of understanding which properties are derived from the atomic arrangement (see the IUCr Online Dictionary of Crystallography, http://reference.iucr.org/dictionary/Main_Page). The scientific speciality has far reaching applications in the disciplines of chemistry, biology, physics, mineralogy, material sciences *etc.* In principle, with such a well defined speciality, the various sub-disciplines of crystallography should be bridged easily. However, in practice we find that chemical crystallography (CX) is vastly different from macromolecular crystallography (MX). Variations of specialized software have developed independently for either CX or MX, data formats are often incompatible and misnomers have arisen over time, which all result in confusion and hence hinder the interoperable use within these sub-disciplines. The availability and interoperable use of data is a leading initiative of the Committee on Data of the International Council for Science (CODATA; http://www.codata.org), which highlighted the theme at the International Data Week in Gaborone, Botswana during November 2018. The Cambridge Structural Database (CSD; Groom *et al.*, 2016 ▸) has now reached 1 million deposited structures and the Protein Data Bank (PDB; Berman *et al.*, 2000 ▸) recently celebrated their 150 000th macromolecular structure. Yet, despite the discipline’s maturity, the interoperability of data remains a challenge, as described from the perspective of the CSD in Liebeschuetz *et al.* (2012 ▸) on ligand geometry distortion. Our overview here wishes to assist crystallographers to bridge the disciplines of CX and MX by highlighting key obstacles when including small-molecule organometallic complexes in proteins as well as specifying the software that can accommodate this conversion.

Our research investigates the development of pharmaceuticals containing a radioactive metal centre, specifically rhenium-188/186 and technetium-99m for the nuclear medical treatment and imaging of cancer (Brink *et al.*, 2014 ▸; Alberto, 2018 ▸; Mokolokolo *et al.*, 2018 ▸; Frei *et al.*, 2018 ▸; Brink & Helliwell, 2017 ▸, 2019 ▸). The ideal is to create radioactive complexes that specifically bind to sites linked to human pathologies (Liu, 2004 ▸). This allows for maximum treatment or imaging efficacy of the disease tissue with minimum radiation damage to healthy tissue. Extensive searching of the CSD was conducted to help interpret crystallographic aspects such as coordination denticity to overcome limits in resolution experienced in MX (Taylor & Wood, 2019 ▸) and utilized the principles of fragment-based drug design. The FBDD approach screens chemical fragments to observe where interactions may occur within the protein (Joseph-McCarthy *et al.*, 2014 ▸; Erlanson, 2012 ▸; Murray *et al.*, 2012 ▸). The information is combined to construct a new ‘chemical complex’. These are research specialities where the interoperability of crystallography becomes vital. The development of small, active ‘lead’ compounds is the domain of chemistry and chemical crystallography, but the coordination within proteins is in the domain of biochemistry and protein crystallography. An overview of knowledge exchange between academic and industry was described by Blundell (2017 ▸) for organic fragments. However, bridging the interoperable region becomes particularly difficult when using fragments containing transition metal elements (*d* or *f* block), elements not commonly found in high concentration in biological organisms.

The breakdown in the interoperability of the two crystallographic specialities can be associated with various aspects (such as misnomers, terminology, software, data formats, precision) using parameters developed specifically for CX or MX.. We will now highlight these differences.

## 2. Databases: internal validation and peer review   

The ability to access data for either CX or MX is an aspect which works well. In CX the databases are dominated by the CSD, the Crystallography Open Database (COD, http://www.crystallography.net/cod/; Gražulis *et al.*, 2009 ▸), and the Inorganic Crystal Structure Database (ICSD; http://www.fiz-karlsruhe.de/icsd.html). For MX, the PDB (now the Worldwide Protein Data Bank or wwPDB) was established in 1971. Other databases are available for powder diffraction, mineralogy, nucleic acids *etc.* and a detailed review is available describing these resources (Bruno *et al.*, 2017 ▸). The data centres should be commended for abiding by the data principles of FAIR (findable, accessible, interoperable and reusable). A critical factor in crystal structure data analysis is the validation process, as individual research results and published data must be held to a standard to prove validity. For chemical crystallography, the IUCr CheckCIF webserver (http://journals.iucr.org/services/cif/checking/checkfull.html) compares small molecules to an absolute standard, yielding a CheckCIF report with Alerts A, B, C or G which must be addressed before deposition of the structure to the database. It is common, if not compulsory practice to provide editors and journal reviewers with the refined data (.ins, .hkl, .fcf, .cif files) as well as the CheckCIF report whenever a manuscript is submitted.

The wwPDB Validation Service (Young *et al.*, 2017 ▸), similarly provides an MX validation report, indicating a relative standard whereby the pdb file is compared with all deposited pdb files at a specific resolution. Errors, deviations from the norm and clashes are indicated in the report, which the authors can address or discuss as necessary. For macromolecular crystallography it is not yet standard practice to automatically provide the refined pdb data files to the reviewers upon submission which can hinder a complete peer-review process (Helliwell, 2018 ▸). Also, data is often requested to be deposited at the wwPDB Deposition Server before the publication review process has begun. If a reviewer requests a correction, it requires a recall and correction to the already deposited structure. However, the practice of submitting various versions of a structure has recently been introduced by the wwPDB.

## 3. Terminologies: the first breakdown of interoperability   

One of the first breakdowns in interoperability between CX and MX is the use of different definitions. In coordination chemistry as well as CX a ‘ligand’ is an ion or molecule (a functional group) that binds to a central metal atom to form a coordination complex. In biochemistry it is a substance that forms a complex with a biomolecule to serve a biological purpose. In protein-ligand binding, the ligand is usually a molecule that produces a signal by binding to a site on a target protein. This is in effect the same definition for two very different aspects. The IUPAC fortunately recognizes this possible source of confusion and states: ‘the definition makes it clear that the view of which entity is central may change for convenience.’ As well as stating that ‘biochemists should bear in mind that the usage in inorganic chemistry has been that ligands bind only single atoms, so they should be cautious in fields such as bioinorganic chemistry where confusion may be possible’ (McNaught & Wilkinson, 1997 ▸). To a crystallographer (CX or MX), this definition, and the field in which it is applied, is rarely clearly listed and confusion is experienced when inter-disciplinary research is conducted.

**Figure 1:**
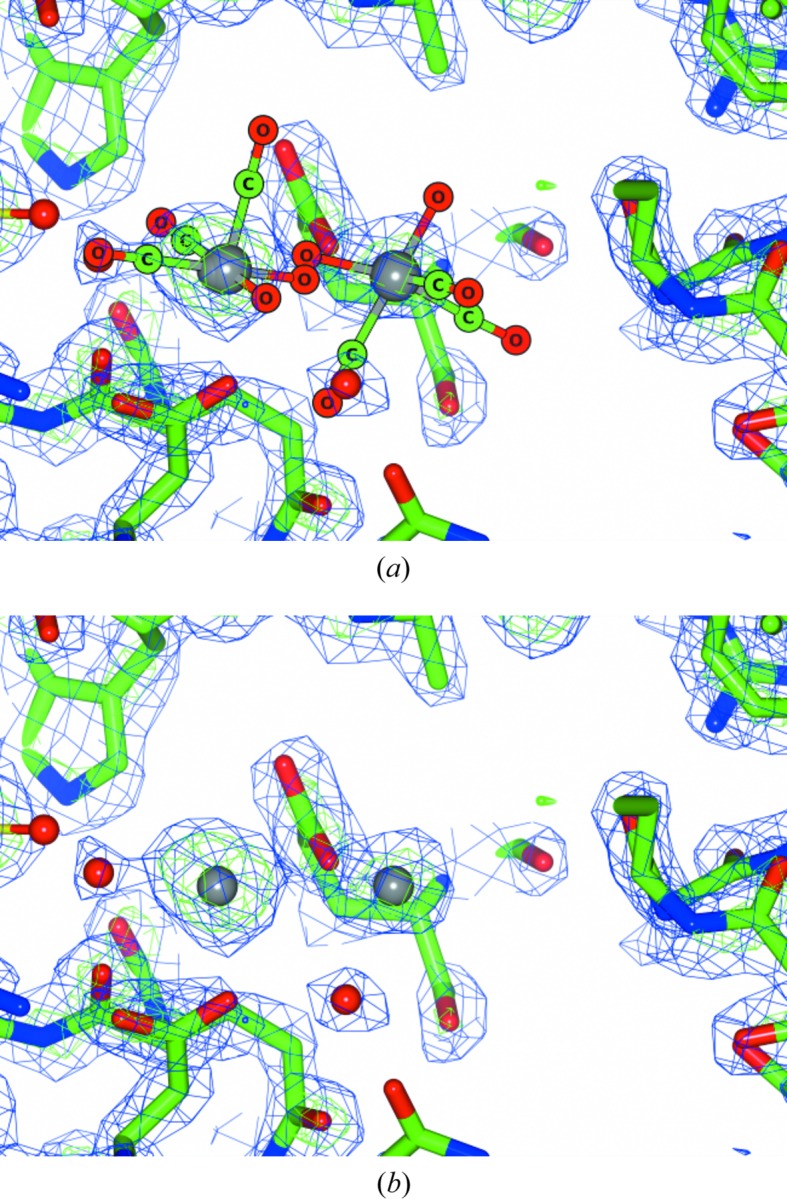
The challenge of resolution and interoperable usage in respective disciplines. The question is which is more correct when utilizing cross-discipline scientific fields? Is a chemically correct representation (*a*) indicating the small molecule 3D structure which is known from the literature, alternative supporting information and significant kinetic understanding of the reaction mechanisms (Roodt *et al.*, 2011 ▸) the more accurate representation? Or is a protein crystallographic representation (*b*) more correct, only displaying atoms with resolvable electron density, but which is not illustrative of the full chemical structure because of the lower resolution obtained in MX? Reproduced from Brink & Helliwell (2017 ▸).

Interoperable differences also occur with regards to crystallographic terminology. Resolution is the ability to distinguish neighbouring features in an electron-density map. In MX the term is commonly used as macromolecular structures vary considerably from 0.8 to 3.0 Å resolution. The higher the resolution (with low numerical value), the better. In CX, the term is rarely considered, unless charge-density studies are being conducted, as the data resolution is always high – at atomic resolution. An illustration of the effects of resolution on what is ‘chemically’ more correct versus ‘macromolecular crystallographically’ more correct in interdisciplinary research is shown in Fig. 1 ▸. It raises the question: does the crystallographer include ligands and residue chains in a structure which chemically must be present even when crystallographically there is insufficient resolution to indicate electron density? The ‘chemically’ more correct understanding must naturally be supported by other experimental information (*i.e.* IR, NMR, mass spectrometry *etc*.). We undertook a compromise for this CX versus MX resolution dilemma, namely submitting the ‘crystallographically acceptable’ PDB deposited data with ‘naked’ rheniums (no ligands) and attaching the ‘more chemically correct’ coordinates file of protein with ligand data as supplementary data to the published manuscript (Fig. 1 ▸). Hence, the reader can obtain appropriate descriptions from both research disciplines.

Another break in terminology usage are the parameters of *B* factors used in MX and anisotropic displacement parameters (ADP) used in CX. Chemical crystallography speaks primarily of ADP, the displacement (including thermal motion and/or disorder) of each atom. The atomic resolution of CX is high and therefore six parameters can be used to describe the displacement. In MX the *B* factor or isotropic refinement also describes the degree of electron-density spread, however it is described by a single parameter as a result of the resolution usually being lower. A good description of *B* factors is discussed by Merritt (2012 ▸). In CX, the ADP is best understood visually using graphical software (*i.e.* *ORTEP* by Farrugia, 2012 ▸) whereas the use in MX is best understood numerically.

## 4. Interoperability barrier two: electron-density maps versus peak list   

The usage of electron-density maps is distinct between MX versus CX. In CX the resolutions obtained are so high that refinement is less dependent on the crystallographer’s ability to interpret the maps. Software which has dominated small-molecule refinement, *i.e.* *WinGX* and *SHELX* (Sheldrick, 2008*a* ▸, 2015 ▸), simplifies the electron-density maps to *q* peaks which represent peaks and troughs. In MX the crystallographer utilizes various maps, such as *F*_o_ − *F*_c_ (omit or difference map), 2*F*_o_ − *F*_c_ and anomalous difference density maps. The integrated use of electron-density maps in CX and MX is slowly becoming a reality, with the development of CX software such as *OLEX 2* (Dolomanov *et al.*, 2009 ▸) which utilizes both *q* peaks and electron-density peaks extensively as well as the use of Tim Grüne’s *shelx2map* (http://shelx.uni-ac.gwdg.de/~tg/research/programs/conv/shelx2map/), which can convert CX .fcf files into a *CCP*4 format map that is compatible with *Coot* (Emsley *et al.*, 2010 ▸). Other critical differences between CX and MX are highlighted in the paper by Groom & Cole (2017 ▸) which is a valuable resource for promoting interoperable crystallographic usage, in particular, the way that organic CX molecules can be used to complement protein-ligand structures for drug discovery.

## 5. Interoperability barrier three, a very big one currently: file formats   

The use of various file formats is perhaps the largest hindrance to interoperability between CX and MX. The IUCr has driven the development of the Crystallographic Information File, CIF, which holds all the crystallographic information of a structure. It is based on a STAR file structure and lists data values, looped together. The structure and development of the CIF file is well summarized by Hall *et al.* (1991 ▸), Brown & McMahon (2002 ▸) and Bernstein *et al.* (2016 ▸). What is important to note is that each line, word, space, comma or semi-colon has a specific meaning. Therefore, any change alters the structure, and hence usability, of the CIF file, and as chemical crystallographers well know when finalizing a cif document for submission, nothing is more frustrating than deleting a semi-colon and then having to find it again. For small molecules, the use of the IUCr’s *publCIF* software greatly assists CIF preparation.

The file formats used in CX and MX for refinement and data submission are distinct. In CX, software generally utilizes an instruction file (.ins) which generates a results file (.res) after each refinement containing the atomic coordinates. The .hkl file contains the experimental derived reflection data, which is converted into a .fcf file, a more compatible CIF reflection file. These files are finally combined into a .cif file suitable for data submission. (Note that *Olex2* utilizes a metaCIF file for refinement, however the .ins and .res files are still independently accessible similarly to that used in *SHELX*.) Standard practice now specifies that all these components are included in the final CX .cif file. Hence, downloading a new .cif file allows the crystallographer to refine data from other researchers, while previously only the structural data were provided.

In MX, the atomic coordinates were traditionally listed in the .pdb file, whereas the reflection data/structure factors were in the .mtz file. These two separate files were submitted to the wwPDB or combined into an mmCIF file. From July 2019, this practice has been discontinued and PDBx/mmCIF format files are now mandatory for MX depositions to the wwPDB.

However, remember that in crystallographic information files, each data line and data loop has a specific format. Hence, if the format changes, the data transferred changes. The formats of the CX .ins, .fcf, .hkl and .cif are vastly different from MX .pdb, .mtz and .mmCIF files, and therefore are processed by software very differently. A perusal of CX .cif files versus the MX .pdb files will immediately allow the reader to identify the variations. Development of interoperable software able to extract data from both CX and MX is therefore commended.

Our research interest is the refinement of small molecular organometallic (emphasis on the organometallic) fragments in proteins, and requires the interoperable use of software and data extraction in both CX and MX. The refinement of small molecular coordinates in protein software remains a challenge. The refinement of a small-molecule *organic* compound in a protein is simply conducted. A monomer ligand CIF library is available in *Coot* (Emsley *et al.*, 2010 ▸; Emsley & Cowtan, 2004 ▸), *CCP*4 (Winn *et al.*, 2011 ▸) and *PHENIX* (Adams *et al.*, 2010 ▸) with common ‘ligands’ (note ‘ligand’ as defined by a biochemist). It is fairly straightforward to generate a unique monomer CIF file. The problem arises when generating an organometallic ‘ligand’ CIF file, with specific metal configuration obtained either from CX structural data or computation calculations. Visualization software is commendably interoperable, but the usage of refinement software tends to result in failed calculations. Also note that the MX software designed for organic atoms struggles with the refinement of organometallic complexes, particularly dense metallic clusters with high electron density. We have utilized *SHELXL*, *PHENIX* and *CCP*4, and each of these struggles with defining the electron-density map surrounding the metallic clusters. *SHELXL* copes the best with the refinement, but unfortunately the format seems not to be compatible with the current PDB validation report server, an aspect still under discussion. The generation of the organometallic ‘ligand’ monomer CIF file which is compatible and refineable in a protein structure has been our stumbling block, and therefore we suggest the following ways to circumvent the challenge.

Small simplistic complexes with *ca* five atoms are best refined freely. Monomer CIF files of complexes which are nearly organic in nature (*i.e.* 1st and 2nd row periodic elements) can be created by utilizing *Sketcher* or *LibCheck*, or by converting the file formats stepwise using *Mercury* (Macrae *et al.*, 2008 ▸), *XPREP* (Sheldrick, 2008*b* ▸) and then *SHELXL* (Sheldrick, 2015 ▸). An excellent resource for additional software options found in *CCP*4 is described by Nicholls (2017 ▸). Organometallic complexes can be made using *JLigand* (Lebedev *et al.*, 2012 ▸), drawn using the SMILES notation. Caution is needed, as the organometallic configuration, particularly around the transition metals, tends to be inaccurate. Bond angles, distances and particularly the torsion angles will have to be defined by script modification and an understanding of chemical configuration. For larger organometallic complexes this approach tends to be tedious. Our best recommendation for the conversion of small-molecule organo­metallic CIF coordinates to a compatible monomer CIF file is to utilize *Mercury*, followed by *eLBOW* from *PHENIX* and *REEL* (Moriarty *et al.*, 2009 ▸, 2017 ▸). The organo­metallic configuration must be corrected in *REEL*, but this is a straightforward exercise. After which the protein-organometallic compound can be refined in *PHENIX* followed by *CCP*4 if required.

Other software which we have found to be interoperable are listed below. Please note that these are our personal favourites with which we were able to accommodate CX organometallic complexes. Conversion of CX file formats to that suitable for protein refinement can be conducted using *Mercury* (.ins; .res; .cif to .pdb). *Diamond* (Putz & Brandenburg, 2014 ▸) is useful for conversion of theoretical structures calculated using *GAUSSIAN* (Frisch *et al.*, 2004 ▸) and CX .cif to .pdb and *MDL* .mol files. Current pdb files can be viewed in *Diamond* (but significant computing power is required) or by using *Mercury*. *XPREP* is very versatile for converting various CX files to MX files and vice versa. The file format generated is occasionally old style hence not always acceptable for the PDB validation check, and may require visual inspection and modification.

## 6. Interoperability barrier four: failure to understand non-covalent distance precision in protein crystallography   

Finally, drug development based on structure information has the potential of becoming as accurate as an architectural construction, however correct analysis and then a proper description of precision is critical (Cooper *et al.*, 2011 ▸). It is deceptive in MX that the displayed bond distance (often visualized in *Coot*) is indicated with the precision to two decimal places without considering the standard deviation which would occur because of resolution, completeness, *B* factors *etc.* In CX, precision is automatically calculated and clearly noted in the various software programs such as *Mercury*, *Diamond* and *Olex2*. Cruickshank (1999 ▸) led the way for protein crystallography to have an understanding of non-covalent distance precision, but its adoption in publications is patchy. However, for MX this precision **must be** determined and can be calculated with the online Diffraction Precision Index (DPI; http://cluster.physics.iisc.ernet.in/dpi/). The calculation of the error on bond distances and angles is described in the papers by Kumar *et al.* (2015 ▸) and Gurusaran *et al.* (2014 ▸). Once protein–organometallic refinement is successful and precision is obtained, then a wealth of information can be harvested for structural drug development utilizing the advantages of both MX and CX with the respective databases of the wwPDB and CSD, in particular the software which has been developed by the CSD such as *Mercury*, *Mogul* (Bruno *et al.*, 2004 ▸), *CrossMiner* and *Gold* (Jones *et al.*, 1997 ▸).

## 7. Conclusions   

Drug development can be approached from numerous perspectives especially when including the use of transition metal complexes. While structure analysis is fundamental to drug development, the interoperability of chemical and biological crystallographic data is a challenge to academic research. The merging of chemical and biochemical data with crystallographic refinement is a powerful option that lies ahead, but will need the interoperability challenges between CX and MX to be overcome. Our organometallic–protein research has highlighted key differences including variations in terminologies, resolution, software, file formats and precision which are stumbling blocks for crystallographic interdisciplinary research that can be overcome by the cross usage of interoperable software listed in this article.

## References

[bb1] Adams, P. D. *et al.* (2010). *Acta Cryst.* D**66**, 213–221.

[bb2] Alberto, R. (2018). *J. Organomet. Chem.* **869**, 264–269.

[bb52] Berman, H. M., Westbrook, J., Feng, Z., Gilliland, G., Bhat, T. N., Weissig, H., Shindyalov, I. N. & Bourne, P. E. (2000). *Nucleic Acids Res.* **28**, 235–242. 10.1093/nar/28.1.235 PMC102472 10592235

[bb3] Bernstein, H. J., Bollinger, J. C., Brown, I. D., Gražulis, S., Hester, J. R., McMahon, B., Spadaccini, N., Westbrook, J. D. & Westrip, S. P. (2016). *J. Appl. Cryst.* **49**, 277–284.

[bb4] Blundell, T. L. (2017). *IUCrJ*, **4**, 308–321. 10.1107/S2052252517009241 PMC5571795 28875019

[bb7] Brink, A. & Helliwell, J. R. (2017). *IUCrJ*, **4**, 283–290. 10.1107/S2052252517003475 PMC5414402 28512575

[bb6] Brink, A. & Helliwell, J. R. (2019). *IUCrJ*, **6**, 695–702. 10.1107/S2052252519006651 PMC6608631 31316813

[bb5] Brink, A., Visser, H. G. & Roodt, A. (2014). *Inorg. Chem.* **53**, 12480–12488. 10.1021/ic5019168 25393647

[bb8] Brown, I. D. & McMahon, B. (2002). *Acta Cryst.* B**58**, 317–324. 10.1107/s0108768102003464 12037350

[bb10] Bruno, I. J., Cole, J. C., Kessler, M., Luo, J., Motherwell, W. D. S., Purkis, L. H., Smith, B. R., Taylor, R., Cooper, R. I., Harris, S. E. & Orpen, A. G. (2004). *J. Chem. Inf. Comput. Sci.* **44**, 2133–2144. 10.1021/ci049780b 15554684

[bb9] Bruno, I., Gražulis, S., Helliwell, J. R., Kabekkodu, S. N., McMahon, B. & Westbrook, J. (2017). *Data Sci. J.* **38**, 1–17.

[bb13] Cooper, D. R., Porebski, P. J., Chruszcz, M. & Minor, W. (2011). *Exp. Opin. Drug. Discov.* **6**, 771–782. 10.1517/17460441.2011.585154 PMC3138648 21779303

[bb15] Cruickshank, D. W. J. (1999). *Acta Cryst.* D**55**, 583–601. 10.1107/s0907444998012645 10089455

[bb16] Dolomanov, O. V., Bourhis, L. J., Gildea, R. J., Howard, J. A. K. & Puschmann, H. (2009). *J. Appl. Cryst.* **42**, 339–341.

[bb19] Emsley, P. & Cowtan, K. (2004). *Acta Cryst.* D**60**, 2126–2132. 10.1107/S0907444904019158 15572765

[bb18] Emsley, P., Lohkamp, B., Scott, W. G. & Cowtan, K. (2010). *Acta Cryst.* D**66**, 486–501. 10.1107/S0907444910007493 PMC2852313 20383002

[bb20] Erlanson, D. A. (2012). *Top. Curr. Chem.* **317**, 1–32. 10.1007/128_2011_180 21695633

[bb21] Farrugia, L. J. (2012). *J. Appl. Cryst.* **45**, 849–854.

[bb22] Frei, A., Mokolokolo, P. P., Bolliger, R., Braband, H., Tsosane, M. T., Brink, A., Roodt, A. & Alberto, R. (2018). *Chem. Eur. J.* **24**, 10397–10402. 10.1002/chem.201800600 29672957

[bb17] Frisch, M. J. *et al.* (2004). *GAUSSIAN03*, Revision C.01, Gaussian Inc., Wallingford, CT, USA.

[bb55] Gražulis, S., Chateigner, D., Downs, R. T., Yokochi, A. F. T., Quirós, M., Lutterotti, L., Manakova, E., Butkus, J., Moeck, P. & Le Bail, A. (2009). *J. Appl. Cryst.* **42**, 726–729. 10.1107/S0021889809016690 PMC3253730 22477773

[bb54] Groom, C. R., Bruno, I. J., Lightfoot, M. P. & Ward, S. C. (2016). *Acta Cryst.* B**72**, 171–179. 10.1107/S2052520616003954 PMC4822653 27048719

[bb23] Groom, C. R. & Cole, J. C. (2017). *Acta Cryst.* D**73**, 240–245. 10.1107/S2059798317000675 PMC5349436 28291759

[bb25] Gurusaran, M., Shankar, M., Nagarajan, R., Helliwell, J. R. & Sekar, K. (2014). *IUCrJ*, **1**, 74–81. 10.1107/S2052252513031485 PMC4104967 25075321

[bb26] Hall, S. R., Allen, F. H. & Brown, I. D. (1991). *Acta Cryst.* A**47**, 655–685.

[bb27] Helliwell, J. R. (2018). *Crystallogr. Rev.* **24**, 263–272.

[bb32] Jones, G., Willett, P., Glen, R. C., Leach, A. R. & Taylor, R. (1997). *J. Mol. Biol.* **267**, 727–748. 10.1006/jmbi.1996.0897 9126849

[bb31] Joseph-McCarthy, D., Campbell, A. J., Kern, G. & Moustakas, D. (2014). *J. Chem. Inf. Model.* **54**, 693–704. 10.1021/ci400731w 24490951

[bb33] Kumar, K. S. D., Gurusaran, M., Satheesh, S. N., Radha, P., Pavithra, S., Thulaa Tharshan, K. P. S., Helliwell, J. R. & Sekar, K. (2015). *J. Appl. Cryst.* **48**, 939–942.

[bb34] Lebedev, A. A., Young, P., Isupov, M. N., Moroz, O. V., Vagin, A. A. & Murshudov, G. N. (2012). *Acta Cryst.* D**68**, 431–440. 10.1107/S090744491200251X PMC3322602 22505263

[bb35] Liebeschuetz, J., Hennemann, J., Olsson, T. & Groom, C. R. (2012). *J. Comput. Aided Mol. Des.* **26**, 169–183. 10.1007/s10822-011-9538-6 PMC3292722 22246295

[bb36] Liu, S. (2004). *Chem. Soc. Rev.* **33**, 445–461.

[bb37] Macrae, C. F., Bruno, I. J., Chisholm, J. A., Edgington, P. R., McCabe, P., Pidcock, E., Rodriguez-Monge, L., Taylor, R., van de Streek, J. & Wood, P. A. (2008). *J. Appl. Cryst.* **41**, 466–470.

[bb38] McNaught, A. D. & Wilkinson, A. (1997). Editors. *IUPAC Compendium of Chemical Terminology*, 2nd ed. Oxford: Blackwell Scientific Publications.

[bb39] Merritt, E. A. (2012). *Acta Cryst.* D**68**, 468–477. 10.1107/S0907444911028320 PMC3322606 22505267

[bb40] Mokolokolo, P. P., Frei, A., Tsosane, M. S., Kama, D. V., Schutte-Smith, M., Brink, A., Visser, H. G., Meola, G., Alberto, R. & Roodt, A. (2018). *Inorg. Chim. Acta*, **471**, 249–256.

[bb42] Moriarty, N. W., Draizen, E. J. & Adams, P. D. (2017). *Acta Cryst.* D**73**, 123–130. 10.1107/S2059798316016570 PMC5297915 28177308

[bb41] Moriarty, N. W., Grosse-Kunstleve, R. W. & Adams, P. D. (2009). *Acta Cryst.* D**65**, 1074–1080. 10.1107/S0907444909029436 PMC2748967 19770504

[bb43] Murray, C. W., Verdonk, M. L. & Rees, D. C. (2012). *Trends Pharmacol. Sci.* **33**, 224–232. 10.1016/j.tips.2012.02.006 22459076

[bb44] Nicholls, R. A. (2017). *Acta Cryst.* D**73**, 158–170. 10.1107/S2059798316020143 PMC5297919 28177312

[bb45] Putz, H. & Brandenburg, K. (2014). *Diamond* – *Crystal and Molecular Structure Visualization*. Crystal Impact, GbR, Kreuzherrenstrasse 102, 53227 Bonn, Germany.

[bb46] Roodt, A., Visser, H. G. & Brink, A. (2011). *Crystallogr. Rev.* **17**, 241–280.

[bb47] Sheldrick, G. M. (2008*a*). *Acta Cryst.* A**64**, 112–122. 10.1107/S0108767307043930 18156677

[bb48] Sheldrick, G. M. (2008*b*). *XPREP*, Version 2008/2. Bruker AXS Inc., Madison, USA.

[bb49] Sheldrick, G. M. (2015). *Acta Cryst.* C**71**, 3–8.

[bb50] Taylor, R. & Wood, P. A. (2019). *Chem. Rev.* https://dx.doi.org/10.1021/acs.chemrev.9b00155.

[bb51] Winn, M. D. *et al.* (2011). *Acta Cryst.* D**67**, 235–242.

[bb53] Young, J. Y. *et al.* (2017). *Structure*, **25**, 536–545. 10.1016/j.str.2017.01.004 PMC5360273 28190782

